# Expression of Genes Encoding Multi-Transmembrane Proteins in Specific Primate Taste Cell Populations

**DOI:** 10.1371/journal.pone.0007682

**Published:** 2009-12-04

**Authors:** Bryan D. Moyer, Peter Hevezi, Na Gao, Min Lu, Dalia Kalabat, Hortensia Soto, Fernando Echeverri, Bianca Laita, Shaoyang Anthony Yeh, Mark Zoller, Albert Zlotnik

**Affiliations:** Senomyx, Inc, San Diego, California, United States of America; Duke University, United States of America

## Abstract

**Background:**

Using fungiform (FG) and circumvallate (CV) taste buds isolated by laser capture microdissection and analyzed using gene arrays, we previously constructed a comprehensive database of gene expression in primates, which revealed over 2,300 taste bud-associated genes. Bioinformatics analyses identified hundreds of genes predicted to encode multi-transmembrane domain proteins with no previous association with taste function. A first step in elucidating the roles these gene products play in gustation is to identify the specific taste cell types in which they are expressed.

**Methodology/Principal Findings:**

Using double label in situ hybridization analyses, we identified seven new genes expressed in specific taste cell types, including sweet, bitter, and umami cells (TRPM5-positive), sour cells (PKD2L1-positive), as well as other taste cell populations. Transmembrane protein 44 (TMEM44), a protein with seven predicted transmembrane domains with no homology to GPCRs, is expressed in a TRPM5-negative and PKD2L1-negative population that is enriched in the bottom portion of taste buds and may represent developmentally immature taste cells. Calcium homeostasis modulator 1 (CALHM1), a component of a novel calcium channel, along with family members CALHM2 and CALHM3; multiple C2 domains; transmembrane 1 (MCTP1), a calcium-binding transmembrane protein; and anoctamin 7 (ANO7), a member of the recently identified calcium-gated chloride channel family, are all expressed in TRPM5 cells. These proteins may modulate and effect calcium signalling stemming from sweet, bitter, and umami receptor activation. Synaptic vesicle glycoprotein 2B (SV2B), a regulator of synaptic vesicle exocytosis, is expressed in PKD2L1 cells, suggesting that this taste cell population transmits tastant information to gustatory afferent nerve fibers via exocytic neurotransmitter release.

**Conclusions/Significance:**

Identification of genes encoding multi-transmembrane domain proteins expressed in primate taste buds provides new insights into the processes of taste cell development, signal transduction, and information coding. Discrete taste cell populations exhibit highly specific gene expression patterns, supporting a model whereby each mature taste receptor cell is responsible for sensing, transmitting, and coding a specific taste quality.

## Introduction

Taste receptor cells packaged in taste buds detect sweet, bitter, umami (the savory taste of glutamate), sour, and salty stimuli [1]. Sweet, bitter, and umami G protein-coupled receptors are polarized to apical microvilli where they sample salivary ligands [2], [3]. Sour taste stimuli are sensed by cells expressing the ion channel PKD2L1, a candidate sour taste receptor that complexes with PKD1L3 and is gated by acidic tastants [4]–[6]. Taste receptors are expressed in distinct and non-overlapping taste receptor cell populations; in this manner, each taste quality is recognized by a specialized taste cell type expressing a receptor tuned to that quality [3].

Identification of genes expressed in specific taste cell types is necessary to advance understanding of taste cell function from initial tastant recognition at apical taste receptors, to subsequent activation of signal transduction machinery and second messenger pathways, and concluding with information transfer to gustatory nerve fibers. We recently reported a gene expression database comprised of over 2,300 transcripts present in taste buds but not surrounding lingual epithelial cells in macaques [7]. Using bioinformatics analyses, we identified over two hundred and fifty genes predicted to encode multi-transmembrane domain proteins with no currently known function in taste biology. We focused specifically on multi-transmembrane domain proteins since they may encode novel receptors and ion channels involved in taste signalling and information coding. As a first step towards elucidating the function of these genes in gustation, we performed *in situ* hybridization analyses of this gene set to map transcripts to specific taste cell populations. This report describes the molecular and histological expression profiles of selected genes in both primate and human taste cells. Specific gene products were identified in TRPM5 taste cells, encompassing sweet, bitter, and umami cells, PKD2L1 taste cells, representing sour cells, and other taste cell populations representing candidate immature taste cells polarized toward the bottom of taste buds. A unifying theme of new transcripts identified in TRPM5 cells is their link to calcium signalling processes.

Expression of genes in specific taste cell populations provides novel insights into the processes and pathways active in gustation. Our results illustrate that different populations of taste cells exhibit discrete patterns of gene expression and support a model whereby each taste quality is detected by a specific taste cell population expressing the requisite set of gene products necessary to sense, transmit, and code that particular taste.

## Results

### Identification of Distinct Cell Types by Histology

**Figure 1:**
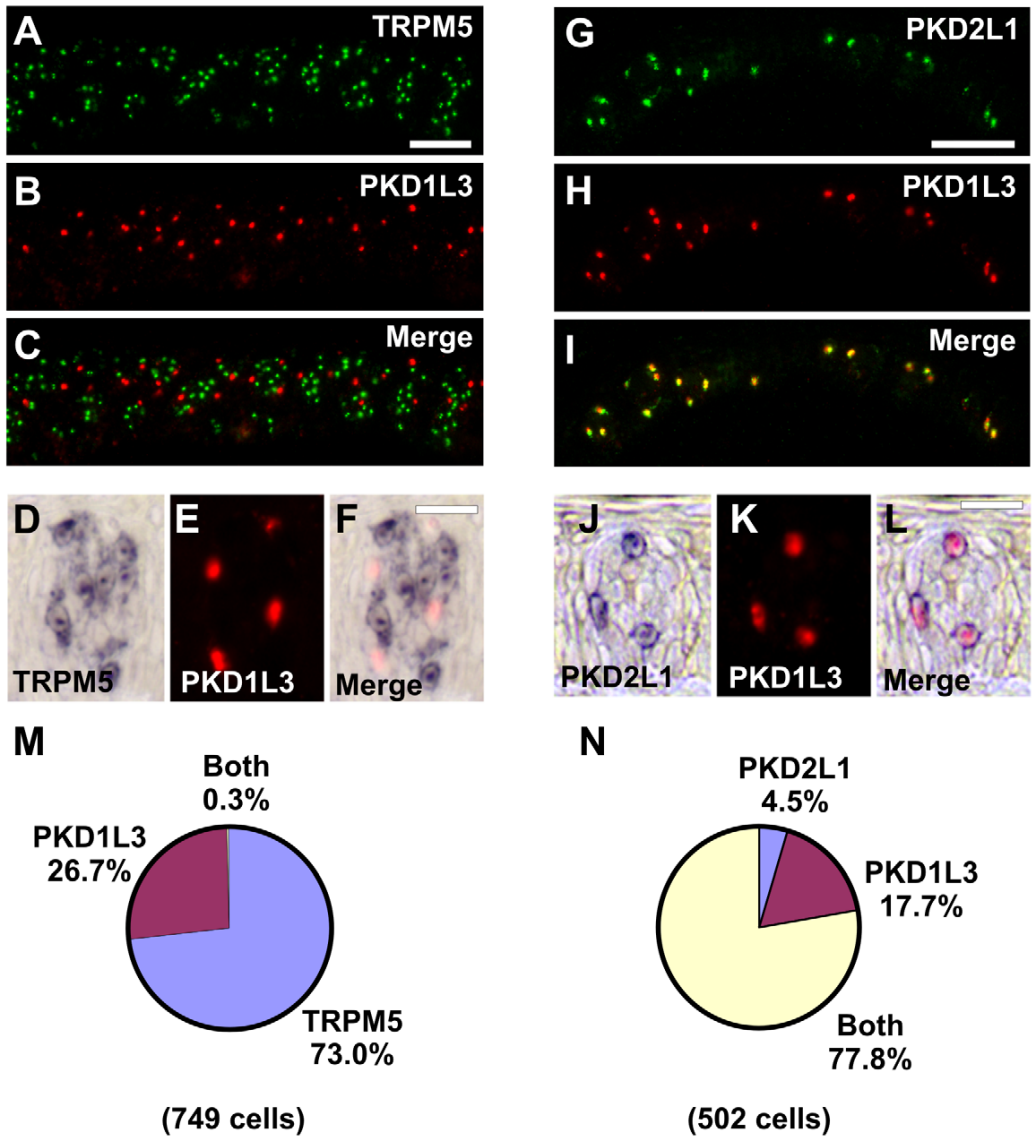
Identification of distinct taste cell populations by histology. A–F, Double label *in situ* hybridization (ISH) for TRPM5 and PKD1L3. TRPM5 (A, D) and PKD1L3 (B, E) are expressed in different cells in the merged images (C, F). G–L, Double label ISH for PKD2L1 and PKD1L3. PKD2L1 (G, J) and PKD1L3 (H, K) are expressed in similar cells in the merged images (I, L). Identical results were obtained using double label fluorescent ISH (A–C and G–I) or double label colorimetric-fluorescent ISH (D–F and J–L). Double label fluorescent ISH images show multiple taste buds whereas double label colorimetric-fluorescent ISH images show a single taste bud. Scale bar is 40µm in A and represents scale for A–C and G–I, 20µm in F and represents scale for D–F and J–L. Images are from primate CV papilla. M, Pie chart illustrating fraction of cells expressing TRPM5, PKD1L3, or both TRPM5 and PKD1L3. N, Pie chart illustrating fraction of cells expressing PKD2L1, PKD1L3, or both PKD2L1 and PKD1L3.

To elucidate the expression pattern of genes encoding multi-transmembrane domain proteins in specific taste cell types, we used double label *in situ* hybridization (ISH) to visualize distinct taste cell populations. Taste receptor cells sensing sweet, bitter, and umami taste stimuli express TRPM5, a calcium-activated, monovalent selective cation channel implicated in taste cell depolarization [8]–[10]. Taste receptor cells sensing sour taste stimuli express PKD2L1, an ion channel that binds PKD1L3 and is gated by acidic tastants [4]–[6]. Ablation of PKD2L1 cells selectively inhibits sour taste nerve responses [4]. Therefore, probes for TRPM5 label sweet, bitter, and umami taste cells whereas probes for PKD2L1 label sour taste cells. A surrogate marker for salty taste cells has not been determined. Using double label ISH, TRPM5 and PKD1L3 labeled distinct taste cell populations (Fig. 1A–F,M), whereas PKD2L1 and PKD1L3 largely labeled the same taste cell population (Fig. 1G–L,N). There were an average of 5.5 TRPM5-positive cells, 2.3 PKD1L3-positive cells, and 1.8 PKD2L1-positive cells per taste bud section in these experiments, and each taste bud section contained 12-18 taste cells depending on the plane of section. As PKD2L1 signals were less robust than PKD1L3 signals, we utilized PKD1L3 probes to mark PKD2L1 cells in this study. Identical results were obtained using double label fluorescent ISH (Fig. 1A–C and G–I) or double label colorimetric-fluorescent ISH (Fig. 1D–F and J–L). As TRPM5 and PKD probes labeled roughly half of macaque taste cells, taste buds clearly house additional cell types; these may include support cells, stem cells, developing cells, and cells for other taste modalities including salty taste.

### TMEM44 Is Expressed in Taste Cells Distinct from TRPM5 and PKD1L3 Cells and at the Bottom of Taste Buds

**Figure 2:**
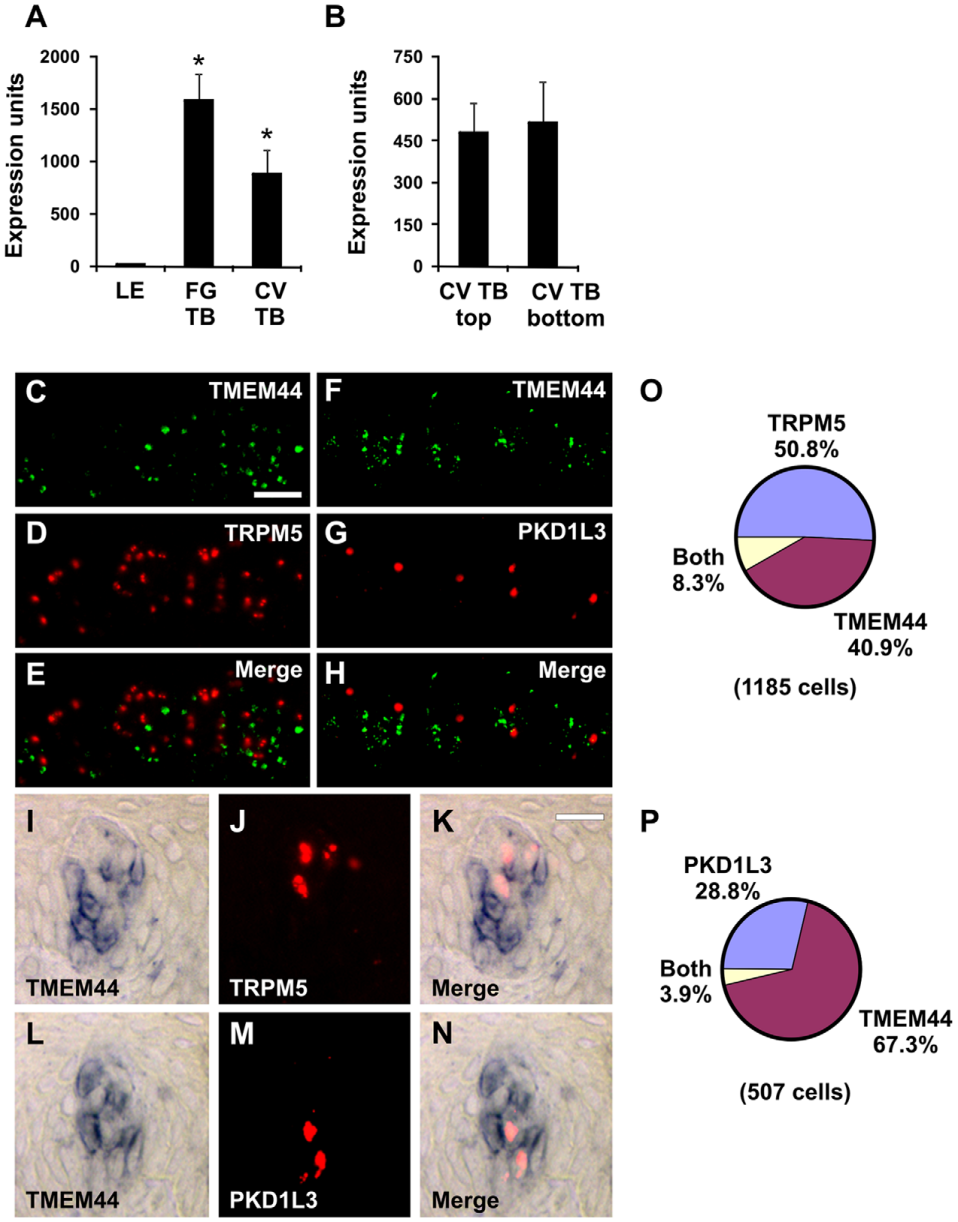
TMEM44 is not expressed in sweet, bitter, umami, and sour cells. Expression of TMEM44 in LE, FG TB, and CV TB (A) as well as top and bottom portions of CV TB (B) by microarray analyses. * p<0.05 compared to LE. Expression units are GC-RMA normalized average intensities of microarray signals. Double label *in situ* hybridization (ISH) for TMEM44 and TRPM5 (C–E and I–K). TMEM44 (C, I) and TRPM5 (D, J) are expressed in different cells in the merged images (E, K). Identical results were obtained in CV (C–E) or FG taste buds (I–K). Double label ISH for TMEM44 and PKD1L3 (F–H and L–N). TMEM44 (F, L) and PKD1L3 (G, M) are expressed in different cells in the merged images (H, N). Identical results were obtained in primate CV (F–H) or FG taste buds (L–N). Scale bar is 30µm in C and represents scale for C–H. Scale bar is 20µm in K and represents scale for I–N. Images are oblique sections with varying orientations from primate CV papilla. O, Pie chart illustrating fraction of cells expressing TMEM44, TRPM5, or both TMEM44 and TRPM5. P, Pie chart illustrating fraction of cells expressing TMEM44, PKD1L3, or both TMEM44 and PKD1L3.

TMEM44 is predicted to encode a seven transmembrane domain protein with an extracellular N-terminus and an intracellular C-terminus, but with no homology to GPCRs. TMEM44 transcripts were highly expressed in both FG and CV cynomolgus macaque (*Macaca fascicularis*) taste buds (Fig. 2A) and also in top and bottom portions of CV taste buds (Fig. 2B) by microarray analyses. There was an average of 4.1 TMEM44-positive cells per taste bud section in single label experiments. Using double label ISH, TMEM44 and TRPM5 labeled distinct taste cell populations in CV (Fig. 2C–E) and FG (Fig. 2I–K) taste buds. TMEM44 and PKD1L3 also labeled distinct taste cell populations in CV (Fig. 2F–H) and FG (Fig. 2L–N) taste buds. Identical results were obtained using double label fluorescent ISH (Fig. 2C–H) or double label colorimetric-fluorescent ISH (Fig. 2I–N). Note that PKD1L3 is detected in FG taste cells in macaque whereas PKD1L3 was not detected in FG taste cells in mouse [4], [5]. Thus, TMEM44 transcripts were not expressed in either TRPM5 or PKD1L3 taste receptor cells (Fig. 2O–P), and TMEM44 cells define a distinct cell population in macaque taste buds.

**Figure 3:**
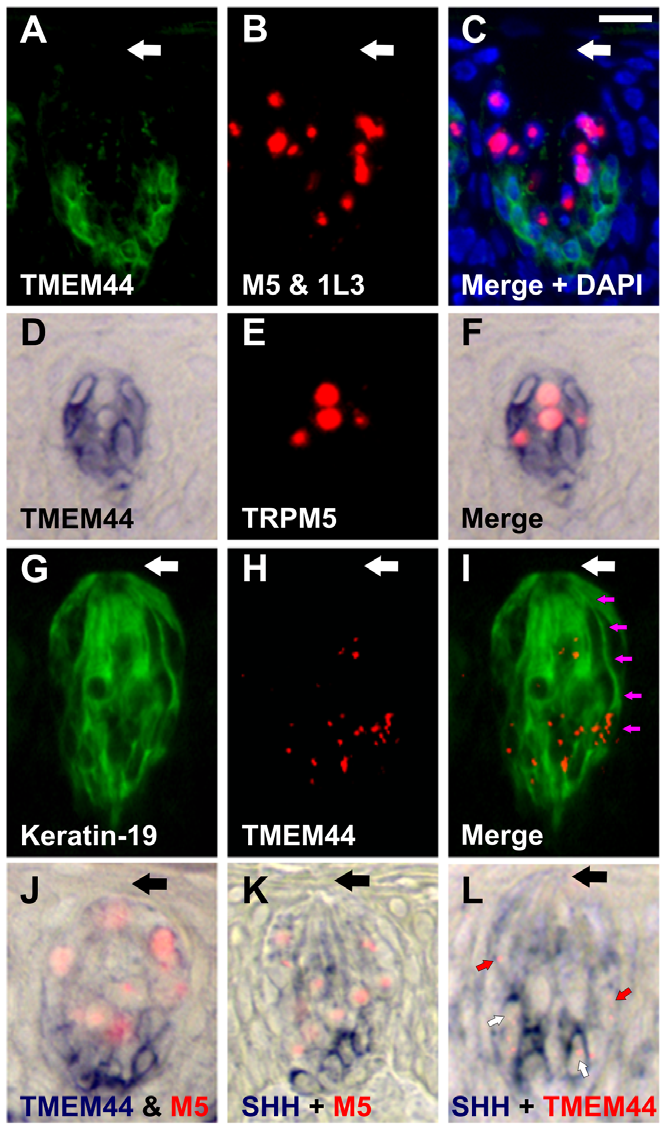
TMEM44 cells localize to the bottom and sides of taste buds. Double label *in situ* hybridization (ISH) for TMEM44 and TRPM5/PKD1L3 in CV taste bud; longitudinal section (A–C). TMEM44 cells (A) are enriched towards the base and sides of taste buds whereas TRPM5/PKD1L3 cells (B) are enriched toward the center and top of taste buds. A merged image with nuclei stained blue (DAPI, C) highlights these signals in a longitudinal section. Double label ISH for TMEM44 and TRPM5 in FG taste bud; tangential section in middle of taste bud (D–F). TMEM44 cells (D) surround TRPM5 cells (E) in merged image (F). Some TMEM44 cells extend processes toward the taste pore (G–I). Double label IHC-ISH for Keratin-19 (G, IHC), TMEM44 (H, ISH) and merge (I) in CV taste bud (longitudinal section). Pink arrows track a TMEM44 cell process towards the taste pore. Double label ISH for TMEM44 (blue) with TRPM5 (red) (J), SHH (blue) with TRPM5 (red) (K), and SHH (blue) with TMEM44 (red) (L) in primate CV taste buds (longitudinal sections). Small white arrows denote cells that express both TMEM44 and SHH transcripts, whereas small red arrows denote cells that express only TMEM44 transcripts. Large arrows denote taste pore region. Note that TMEM44 stain in panel A is a colorimetric signal (DIG-labeled ISH probe) that is pseudocolored green, whereas the TMEM44 stain in panel H is a fluorescent signal (FITC-labeled ISH probe). Colorimetric signals highlight the nuclear envelope and cytoplasm whereas fluorescent signals highlight intranuclear regions. Scale bar is 20µm in C and represents scale for A–L.

Previously, we determined that transcripts for taste receptors and signal transduction components were enriched in the top fraction of CV taste buds while transcripts for cell cycle and extracellular matrix proteins were enriched in the bottom fraction of CV taste buds, consistent with a model in which mature taste receptor cells are located in the top portion while developmentally immature taste cells reside in the bottom portion of CV taste buds [7]. Using longitudinal or tangential sections, TMEM44 signals localized to cells at the bottom and sides of CV (Fig. 3A) and FG (Fig. 3D) taste buds. By contrast, TRPM5 and PKD1L3 signals localized to cells toward the top and center region of CV (Fig. 3B) and FG (Fig. 3E) taste buds. Although TMEM44 cell nuclei are enriched in the bottom portion of CV taste buds (Fig. 3H), some TMEM44 cell processes labeled with keratin-19 (Fig. 3G), a marker of taste bud cells [11], extended to the taste pore region (Fig. 3I). TMEM44 transcripts in these cell processes likely account for TMEM44 expression in the top portion of taste buds by microarray analyses (Fig. 2B). Sonic hedgehog (SHH), a growth factor expressed in progenitor cells and important for cell fate and developmental processes is expressed in immature taste cells at the bottom of taste buds in rodents [12]. TMEM44 cells (Fig. 3J) and SHH cells (Fig. 3K) were both polarized toward the bottom of CV taste buds in macaques. Double label ISH revealed that TMEM44 signals partially colocalized with SHH signals (Fig. 3L) in cells at the bottom of taste buds. In addition, a population of TMEM44 cells that did not express SHH was present above the TMEMM44/SHH-positive cells and towards the lateral region of taste buds (Fig. 3L). These data suggest that TMEM44 may be expressed in cells transiting from an immature (SHH-positive) to a mature (taste receptor-positive) state and may represent an intermediate stage in taste cell development.

### TRPM5 Cells Express Genes Linked to Calcium Signalling: MCTP1, CALHM1-3, and ANO7

**Figure 4:**
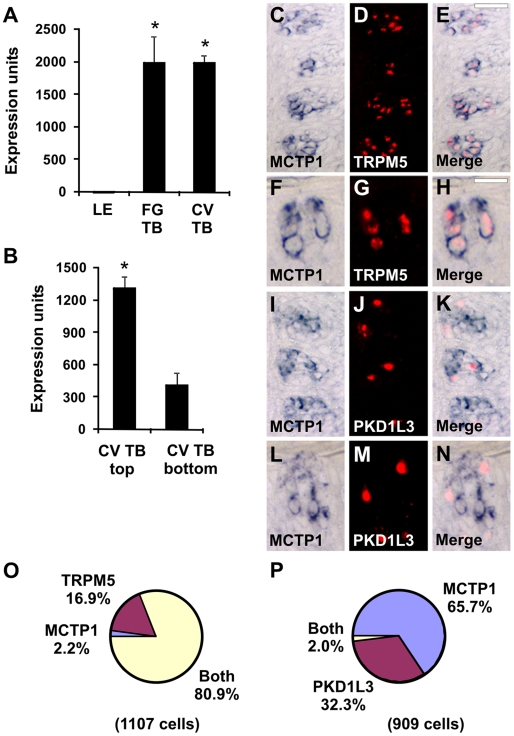
MCTP1 is expressed in TRPM5 cells. Expression of MCTP1 in LE, FG TB, and CV TB (A) as well as top and bottom portions of CV TB (B) by microarray analyses. * p<0.005 compared to LE (A) or CV TB bottom (B). Expression units are GC-RMA normalized average intensities of microarray signals. Double label *in situ* hybridization (ISH) for MCTP1 and TRPM5 (C–H). MCTP1 (C, F) and TRPM5 (D, G) are expressed in similar cells in the merged images (E, H). Double label ISH for MCTP1 and PKD1L3 (I–N). MCTP1 (I, L) and PKD1L3 (J, M) are expressed in different cells in the merged images (K, N). Single taste buds are illustrated in F–H and L–N. Scale bar is 30µm in E and represents scale for C–E and I–K. Scale bar is 25µm in H and represents scale for F–H and L–N. Images are from primate CV papilla. O, Pie chart illustrating fraction of cells expressing MCTP1, TRPM5, or both MCTP1 and TRPM5. Cells with only TRPM5 signals may contain MCTP1 transcripts below the detection limit of ISH. P, Pie chart illustrating fraction of cells expressing MCTP1, PKD1L3, or both MCTP1 and PKD1L3.

**Figure 5:**
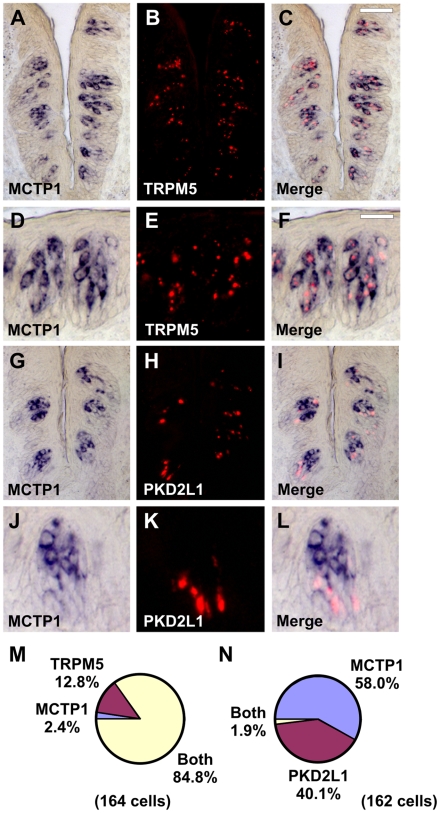
MCTP1 is expressed in TRPM5 cells in mouse. Double label *in situ* hybridization (ISH) for MCTP1 and TRPM5 (A–F). MCTP1 (A, D) and TRPM5 (B, E) are expressed in similar cells in the merged images (C, F). Double label ISH for MCTP1 and PKD2L1 (G–L). MCTP1 (G, J) and PKD2L1 (H, K) are expressed in different cells in the merged images (I, L). Images in D–F and J–L depict single taste buds at higher magnification. Scale bar is 40µm in C and represents scale for A–C and G–I. Scale bar is 25µm in F and represents scale for D–F and J–L. Images are from mouse CV papilla. M, Pie chart illustrating fraction of cells expressing MCTP1, TRPM5, or both MCTP1 and TRPM5. N, Pie chart illustrating fraction of cells expressing MCTP1, PKD2L1, or both MCTP1 and PKD2L1.

MCTP1 is predicted to encode a two transmembrane domain protein with intracellular N- and C-termini, and three calcium-binding C2 domains preceding the first membrane spanning domain [13]. C2 domain-containing proteins are generally involved in signal transduction and membrane trafficking events. MCTP1 transcripts were expressed in FG and CV taste buds (Fig. 4A) and were enriched in the top portion of CV taste buds (Fig. 4B) by microarray analyses. There was an average of 4.7 MCTP1-positive cells per taste bud section in single label experiments. Using double label ISH, MTCP1 and TRPM5 labeled similar taste cell populations (Fig. 4C–H, O) while MCTP1 and PKD1L3 labeled distinct taste cell populations (Fig. 4I–N, P). Identical results were obtained in macaque (Fig. 4) and mouse (Fig. 5), suggesting that MCTP1 function in taste is likely conserved between rodents and primates.

**Figure 6:**
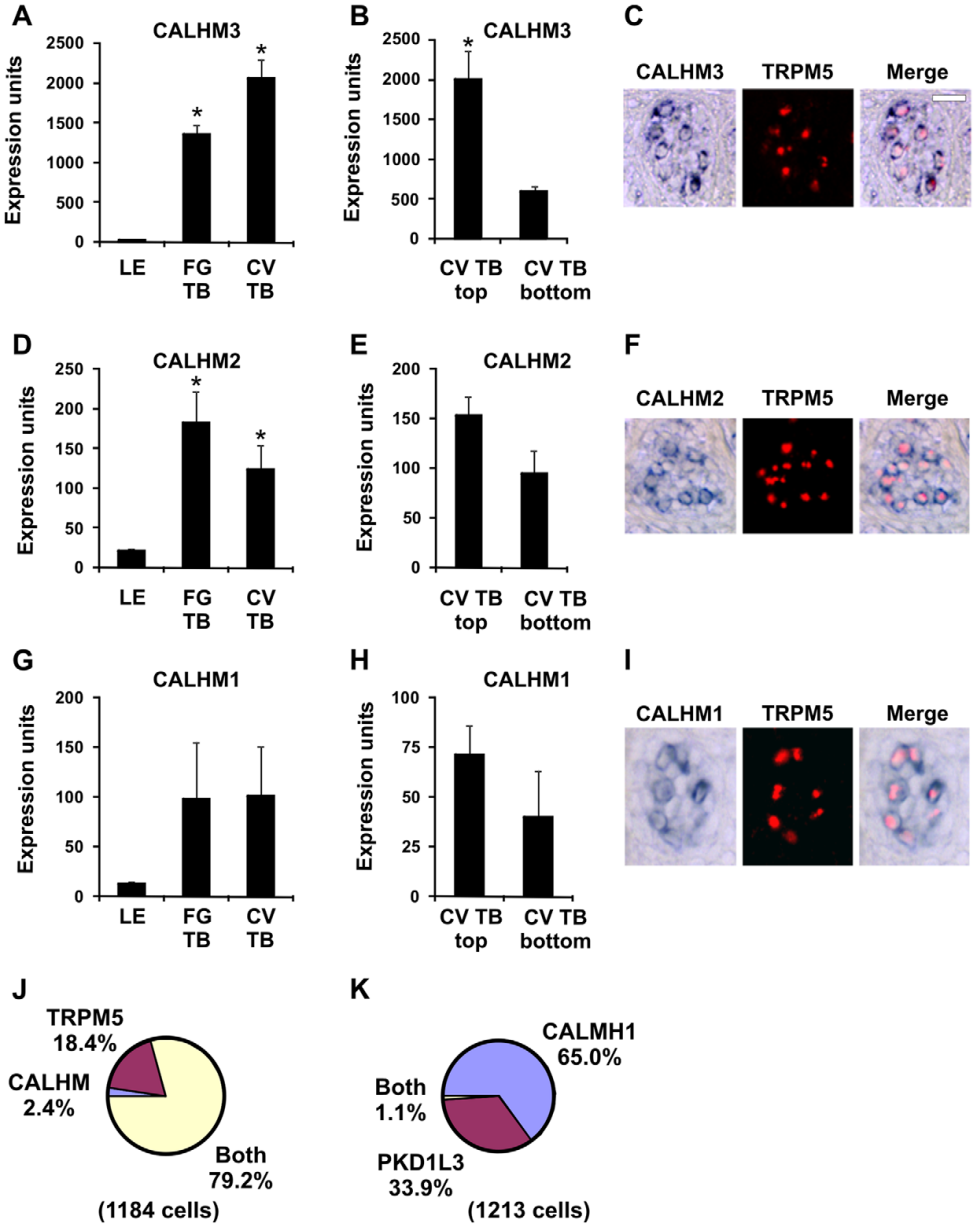
CALHM1, 2, and 3 are expressed in TRPM5 cells. Expression of CALHM3 (A–B), CALHM2 (D–E), and CALHM1 (G–H) in LE, FG TB, and CV TB as well as top and bottom portions of CV TB by microarray analyses. For CALHM3, * p<0.005 compared to LE (A) or p<0.05 compared to CV TB bottom (B); for CALHM2, * p<0.05 compared to LE (D); for CALHM1, p<0.05 for CV top compared to LE. Expression units are GC-RMA normalized average intensities of microarray signals. Double label *in situ* hybridization (ISH) for CALHM3 with TRPM5 (C), CALHM2 with TRPM5 (F), and CALHM1 with TRPM5 (I), illustrating that CALHM 1, 2, and 3 are expressed in TRPM5 cells. Scale bar is 20µm in C and represents scale for C, F, and I. Images are from primate CV papilla. J, Pie chart illustrating fraction of cells expressing CALHM, TRPM5, or both CALHM and TRPM5. Since results were similar for CALHM1, CALHM2, and CALHM3, data were pooled. Individually, there were 2.5% CALHM1 cells, 18.1% TRPM5 cells, and 79.4% of cells expressing both CALHM1 and TRPM5 cells; 3.2% CALHM2 cells, 21.5% TRPM5 cells, and 75.3% of cells expressing both CALHM2 and TRPM5; 0.6% CALHM3 cells; 15.1% TRPM5 cells, and 84.5% of cells expressing both CALHM3 and TRPM5. Cells with only TRPM5 signals may contain CALHM transcripts below the detection limit of ISH. P, Pie chart illustrating fraction of cells expressing CALHM1, PKD1L3, or both CALHM1and PKD1L3.

CALHM1 (FAM26C), CALHM2 (FAM26B), and CALHM3 (FAM26A) are predicted to encode four transmembrane domain proteins with intracellular N- and C-termini. CALHM1 was recently reported to be a component of a novel calcium-permeable channel expressed in hippocampal neurons [14]. CALHM3 (Fig. 6A–B), CALHM2 (Fig. 6D–E), and CALHM1 (Fig. 6G–H) were all expressed in FG and CV taste buds with higher levels in the top portion of CV taste buds by microarray analyses. There were an average of 4.6 CALHM1-positive cells, 4.5 CALHM2-positive cells, and 4.7 CALHM3-positive cells per taste bud section in single label experiments. Using double label ISH, CALHM3 (Fig. 6C), CALHM2 (Fig. 6F), and CALHM1 (Fig. 6I) probes labeled TRPM5 taste cells but not PKD1L3 taste cells (Fig. 6J–K). Thus, similar to MCTP1, transcripts from all three CALHM genes were expressed in TRPM5 taste receptor cells.

**Figure 7:**
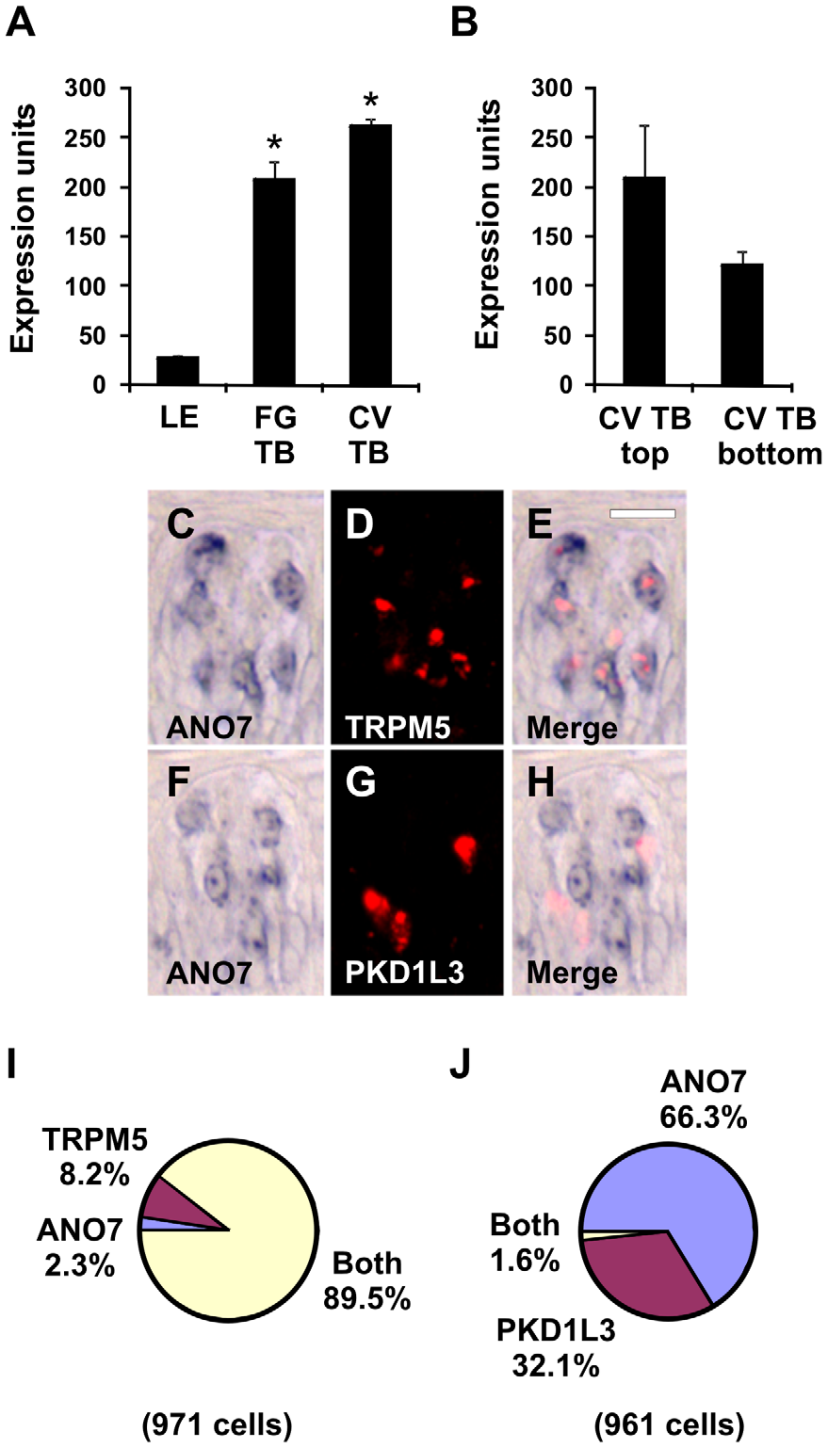
ANO7 is expressed in TRPM5 cells. Expression of ANO7 in LE, FG TB, and CV TB (A) as well as top and bottom portions of CV TB (B) by microarray analyses. * p<0.005 compared to LE (A). Expression units are GC-RMA normalized average intensities of microarray signals. Double label *in situ* hybridization (ISH) for ANO7 and TRPM5 (C–E). ANO7 (C) and TRPM5 (D) are expressed in similar cells in the merged image (E). Double label ISH for ANO7 and PKD1L3 (F–H). ANO7 (F) and PKD1L3 (G) are expressed in different cells in the merged image (H). Images are from primate CV taste buds. Scale bar is 15µm in E and represents scale for C–H. I, Pie chart illustrating fraction of cells expressing ANO7, TRPM5, or both ANO7 and TRPM5. J, Pie chart illustrating fraction of cells expressing ANO7, PKD1L3, or both ANO7 and PKD1L3.

**Figure 8:**
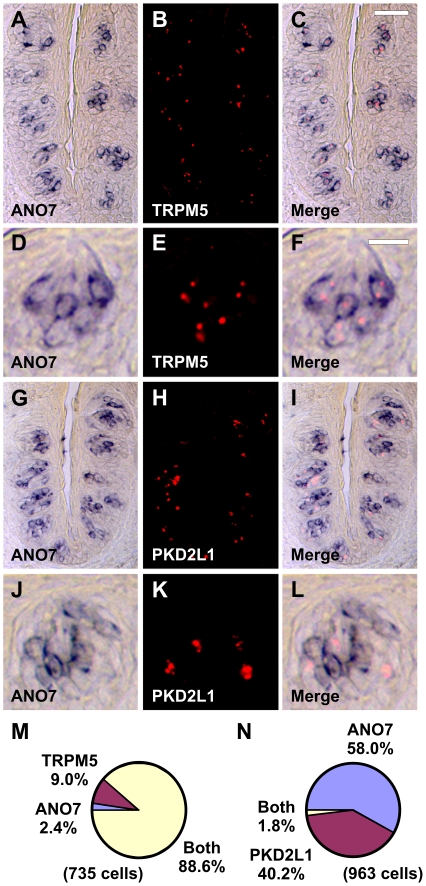
ANO7 is expressed in TRPM5 cells in mouse. Double label *in situ* hybridization (ISH) for ANO7 and TRPM5 (A–F). ANO7 (A, D) and TRPM5 (B, E) are expressed in similar cells in the merged images (C, F). Double label ISH for ANO7 and PKD2L1 (G–L). ANO7 (G, J) and PKD2L1 (H, K) are expressed in different cells in the merged images (I, L). Images in D–F and J–L depict single taste buds at higher magnification. Scale bar is 40µm in C and represents scale for A–C and G–I. Scale bar is 10µm in F and represents scale for D–F and J–L. Images are from mouse CV papilla. M, Pie chart illustrating fraction of cells expressing ANO7, TRPM5, or both ANO7 and TRPM5. N, Pie chart illustrating fraction of cells expressing ANO7, PKD2L1, or both ANO7 and PKD2L1.

ANO7 (TMEM16G) is an eight transmembrane domain protein with intracellular N- and C-termini [15] that belongs to the anoctamin family of newly identified calcium-activated chloride channels [16]. ANO1 (TMEM16A) is a calcium-gated chloride channel in secretory epithelia and retina [17]–[20], while ANO2 (TMEM16B) is a candidate calcium-gated chloride channel in photoreceptor neurons and olfactory sensory neurons [21], . In photoreceptor neurons, ANO2 may modulate pre-synaptic release of glutamate neurotransmitter to second order neurons [22]. In olfactory cilia, ANO2 may mediate efflux of chloride ions following calcium entry through cyclic nucleotide-gated channels, thereby amplifying the magnitude of the depolarizing signal in response to odorants [21], [23]. ANO7 transcripts were expressed in FG and CV taste buds (Fig. 7A) and were more prevalent in the top portion of CV taste buds (Fig. 7B) by microarray analyses. There was an average of 5.2 ANO7-positive cells per taste bud section in single label experiments. Using double label ISH, ANO7 was expressed in TRPM5 cells (Fig. 7C–E) but not PKD1L3 taste cells (Fig. 7F–H, J). ANO7 ISH signals were detectable in most all TRPM5 cells (Fig. 7I), similar to MCTP1 and CALHM1-3 transcripts. ANO7 was also selectively expressed in TRPM5 cells in mouse (Fig. 8), suggesting conservation of ANO7 function in taste between primates and rodents.

Collectively, MCTP1, CALHM1-3, and ANO7 are all expressed with TRPM5 in sweet, bitter, and umami taste receptor cells. These gene products likely participate in the transmission and/or amplification of taste signals (see Discussion).

### SV2B Is Expressed in PKD1L3 Taste Cells

**Figure 9:**
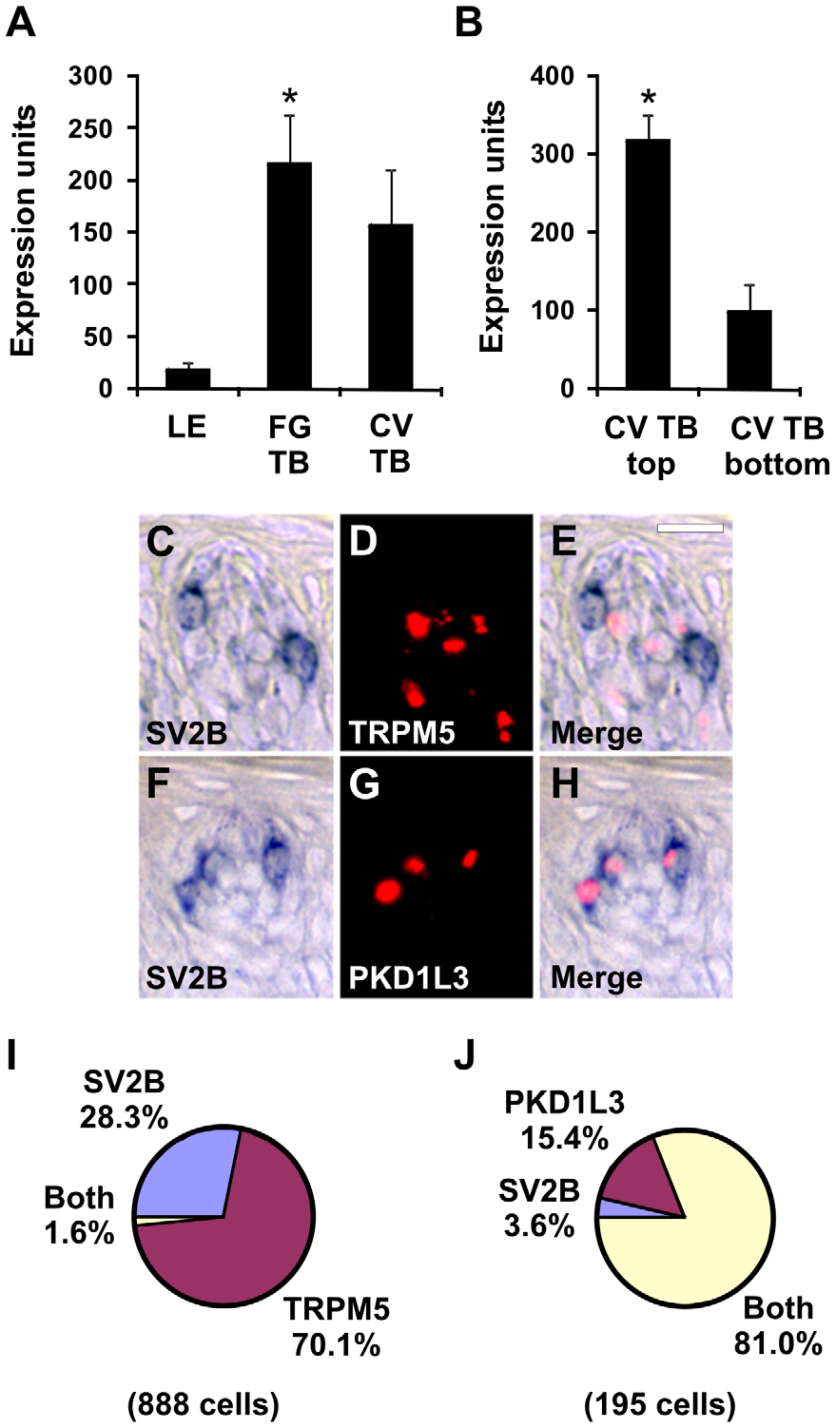
SV2B is expressed in PKD1L3 cells. Expression of SV2B in LE, FG TB, and CV TB (A) as well as top and bottom portions of CV TB (B) by microarray analyses. * p<0.01 compared to LE (A) or CB TB bottom (B). Expression units are GC-RMA normalized average intensities of microarray signals. Double label *in situ* hybridization (ISH) for SV2B and TRPM5 (C–E). SV2B (C) and TRPM5 (D) are expressed in different cells in the merged image (E). Double label ISH for SV2B and PKD1L3 (F–H). SV2B (F) and PKD1L3 (G) are expressed in similar cell types in the merged image (H). Images are from primate CV taste buds. Scale bar is 20µm in E and represents scale for C–H. I, Pie chart illustrating fraction of cells expressing SV2B, TRPM5, or both SV2B and TRPM5. J, Pie chart illustrating fraction of cells expressing SV2B, PKD1L3, or both SV2B and PKD1L3. Cells with only PKD1L3 signals may contain SV2B transcripts below the detection limit of ISH.

SV2B is predicted to encode a twelve transmembrane domain protein with intracellular N- and C-termini, and a major facilitator superfamily permease domain within the transmembrane region. SV2B interacts with synaptotagmin 1, forms a complex with SNARE proteins important in vesicle exocytosis at nerve terminals, and constitutes a receptor for botulinum neurotoxin A [24], [25]. SV2B transcripts were expressed in FG and CV taste buds (Fig. 9A) and were enriched in the top portion of CV taste buds (Fig. 9B) by microarray analyses. There was an average of 2.2 SV2B-positive cells per taste bud section in single label experiments. SV2B and TRPM5 labeled distinct taste cell populations (Fig. 9C–E, I) while SV2B and PKD1L3 expression patterns overlapped (Fig. 9F–H, J), in double label ISH analyses. Thus, SV2B transcripts were expressed in PKD1L3 taste receptor cells.

### Genes Encoding Transmembrane Proteins Are Expressed in Human Taste Buds

**Figure 10:**
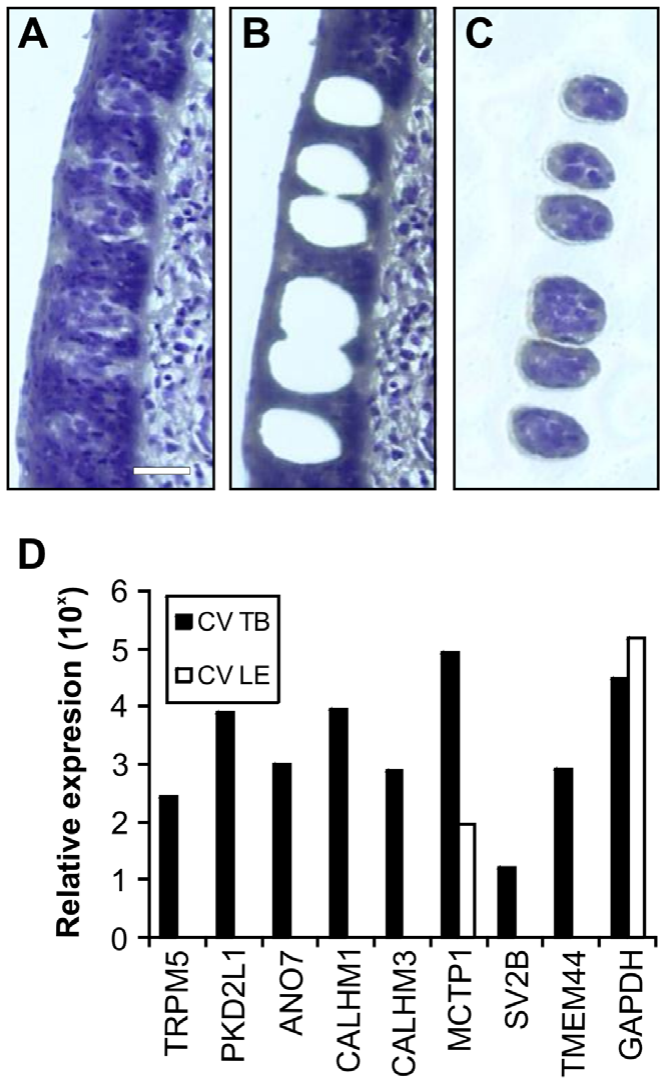
Genes encoding transmembrane proteins are expressed in human CV taste buds. Section of human CV papilla before (A) and after (B) laser capture microdissection of taste buds. Collected taste bud regions (C), were isolated from CV papilla and used for molecular analysis of gene expression. A laser beam was used to cut the perimeter of taste buds and physically separate them from surrounding lingual epithelium. Taste buds were next lifted away from the tissue section with an adhesive cap. Panel C is an image of six isolated taste bud regions, devoid of surrounding lingual epithelium and connective tissue, on the adhesive cap. Scale bar is 40µm. Semi-quantitative PCR (D) for known taste genes (TRPM5 and PKD2L1), genes predicted or known to encode transmembrane proteins, and the housekeeping gene GAPDH in isolated CV taste buds (black bars) or non-gustatory lingual epithelium (white bars) collected by laser capture microdissection. Relative expression is shown on a logarithmic scale.

Human CV taste buds and surrounding lingual epithelium were collected by laser capture microdissection (Fig. 10A–C) and semi-quantitative PCR was performed to determine the expression level of the identified genes. As shown in Fig. 10D, genes encoding transmembrane proteins were expressed in human CV taste bud cells with no detectable or greatly reduced expression in lingual epithelium. These data confirm that taste bud-associated genes identified in macaques are also expressed in human taste buds.

## Discussion

In this study, we describe the molecular and histological expression profiles of seven genes predicted to encode multi-transmembrane domain proteins with no prior association with taste function. These data represent one of the most comprehensive reports of gene expression and transcript localization in macaque and human taste buds to date. A current labeled line model in taste biology contends that each taste quality is recognized by a specialized taste cell type expressing a receptor tuned to that quality; in this manner, each mature taste receptor cell is hard-wired to sense a single taste [3], [26]. Our findings that gene products are preferentially expressed in different taste cell populations with specialized functions supports the labeled line model of gustatory coding in primates whereby differentiated taste cells expresses transcripts necessary to sense, transmit, and code a specific taste quality.

TMEM44 encodes a predicted transmembrane protein that is poorly characterized. TMEM44 is conserved in mammals with 70–80% protein identity between humans and rodents, present in zebrafish and C. elegans genomes, and expressed in diverse tissue types by EST profiling but its function is currently unknown. The closest relative of TMEM44 by sequence alignment, with 25% identity and similar predicted topology, is PQ loop repeat containing 2 (PQLC2), which also has no known function. The TMEM44 amino acid sequence is predicted to contain seven transmembrane domains and does not align to any protein families or domains in the current Pfam database. TMEM44 transcripts localized to taste cells toward the bottom of macaque taste buds that were largely distinct from cells expressing TRPM5 or PKD1L3. TMEM44 cells may comprise a developing taste cell population since immature, basal cells in the bottom of the taste bud express SHH, a growth factor involved in taste bud development [12], [27], [28], and TMEM44 signals partially overlapped with SHH signals. As taste cells mature, they are thought to migrate toward the top region of the taste bud, adopt a spindle-shaped morphology, and start expressing genes for taste receptors and signal transduction components [12]. A small fraction of TMEM44 cells also expressed TRPM5 or PKD1L3 and some TMEM44 cells had apical processes that extended towards the taste pore region, suggesting that these cells may be transitioning from an immature to a mature state.

In addition to the bottom region, TMEM44 cells were also localized to the lateral region of taste buds. In this respect, TMEM44 cells may function, in part, as support cells for taste receptor cells, similar to sustentacular cells that support olfactory sensory neurons in the nasal cavity. A population of taste cells, frequently referred to as type I cells, expressing the glutamate transporter GLAST and the ecto-ATPase NTPDase2 is proposed to clear neurotransmitters, released from TRPM5 and PKD1L3 taste cells, from the extracellular space [29], [30], and cells defined as type I cells by electron microscopic ultrastructural analyses have nuclei located in lateral and bottom region of taste buds [31], [32]. GLAST, encoded by the SCL1A3 gene, was expressed in primate taste buds by microarray analyses, but was not detectable by ISH, while NTPDase2, encoded by the ENTPD2 gene, was not expressed at detectable levels in primate taste buds by microarray analyses (data not shown); thus, we were unable to compare the histological expression pattern of TMEM44 with these gene products in primates.

We identified five genes co-expressed with TRPM5 and predicted to encode transmembrane proteins with links to calcium signalling. Calcium is a crucial signalling molecule in transmitting information from apical taste receptors to basolateral afferent nerve fibers. Following sweet, bitter, and umami taste receptor activation, a signal transduction cascade ensues involving gustducin, phospholipase Cβ2, and type III inositol 1,4,5-triphosphate receptor-mediated release of calcium from intracellular stores. Calcium opens the monovalent cation-selective TRPM5 ion channel and the resultant receptor potential likely triggers activation of voltage-gated sodium channels, firing of action potentials, and information transfer to gustatory nerve fibers [3], [33].

ANO7 is a putative calcium-gated chloride channel or channel subunit expressed in taste buds. The anoctamin gene family comprises 10 members of which two, ANO1 and ANO2 have been demonstrated to function as calcium-gated chloride channels in non-sensory and sensory cells, including photoreceptor and olfactory sensory neurons [17]–[19], [21], [22], [34]. ANO7 exhibits restricted tissue expression and is highly expressed in normal and cancerous prostate where it localizes to apical and lateral membrane regions and may regulate cell aggregation; accordingly ANO7 represents a target for prostate cancer immunotherapy [35]–[37]. We determined that ANO7 is specifically expressed in taste buds and not in surrounding lingual epithelial cells and localized to TRPM5 cells by ISH. In addition to ANO7, transcripts for ANO2, ANO4, ANO5, ANO6, and ANO9 were present in taste buds but signals were not detectable by ISH (data not shown). It would be of great interest to determine the specific role(s) of ANO7 and other anoctamin family members in taste cell biology. Function of a calcium-gated chloride channel in taste receptor cell microvilli at the apical membrane could amplify TRPM5 receptor potentials. As an example, ANO2 is polarized to apical cilia in olfactory sensory neurons and may amplify initial depolarizing signals through CNG channels by mediating chloride efflux into the nasal cavity [21]. Since saliva has a naturally low chloride concentration [38], chloride efflux through apical channels would further depolarize taste receptor cells. Conversely, activation of a calcium-gated chloride channel on basolateral membranes would cause hyperpolarization, due to chloride influx from chloride-replete interstitial fluid around taste cells, and attenuate receptor potentials. As an example, ANO2 is polarized to photoreceptor synaptic terminals in retina and may modulate glutamate release via local control of membrane potential [22]. Notably, calcium-dependent chloride currents that resemble anoctamin currents in their outward rectification and inhibition by DIDS as well as niflumic acid, have been recorded from taste cells [39], [40].

The CALHM gene family (previously called FAM26) comprises 6 members, of which one, CALHM1, has been demonstrated to generate a constitutively active cation channel, with a calcium to sodium permeability ratio of five to one, in heterologous expression systems [14]. CALHM1 along with CALHM2 and CALHM3 were specifically expressed in taste buds and localized to TRPM5 cells by ISH. CALHM proteins have four predicted transmembrane domains and likely define a new ion channel family. Since ion channels generally multimerize to form charge conducting pores, CALHM proteins may homo- or heteromultimerize to generate functional entities to regulate calcium entry from extracellular routes (saliva or interstitial fluid) or release from intracellular stores. Interestingly, a TRPM5-independent cation channel regulated by calcium was observed in taste cells [10]. The relationship of this channel to CALHM proteins requires additional investigation. TRPM5 cells, referred to as type II cells, lack voltage-gated calcium channels [41], [42] and release ATP neurotransmitter by a non-conventional pathway involving pannexin/connexin hemichannels gated by voltage [43]–[45]. Some hemichannels are also gated by intracellular calcium [46]. CALHM proteins may, therefore, provide a conduit for calcium entry leading to hemichannel opening in TRPM5 cells.

The two MCTP genes in vertebrates encode calcium-binding proteins of unknown function [13]. MCTP1 was specifically expressed in taste buds and not surrounding lingual epithelial cells by microarray analyses and localized to TRPM5 cells by ISH. Transcripts for MCTP2 were also present in taste buds but expression was not detected by ISH (data not shown). MCTP1 has three calcium-binding C2 domains in the cytosolic N-terminus. The single MCTP homologue in C. elegans is an essential gene, since RNAi-mediated knockdown was lethal to embryos, supporting a critical function in calcium signalling [47]. C2 domain-containing proteins are generally involved in signal transduction and membrane trafficking events. Thus, MCTP1 could modulate the function and trafficking of taste cell proteins including sweet, bitter, and umami taste receptors or the calcium-gated channel TRPM5. Alternatively, MCTP1 may function as a downstream effector protein following taste receptor activation.

SV2B is a presynaptic vesicle protein important for calcium-regulated neurotransmitter exocytosis [48]–[50]. SV2B localized exclusively to PKD1L3 cells and was not detectable in TRPM5 cells by ISH. Taste cells expressing PKD1L3 and PKD2L1, referred to as type III cells, transmit information to afferent nerve fibers via exocytosis of neurotransmitters [51]–[53]. Expression of SV2B in PKD1L3 cells, along with the synaptic protein SNAP-25 [54], is consistent with transmission of sour taste signals to post-synaptic afferent fibers via conventional synaptic vesicle exocytosis. SV2B represents a receptor for botulinum neurotoxin A [24], a toxin that inactivates SNAP-25 by proteolysis [55], [56]. Botulinum neurotoxin A should therefore selectively inhibit sour taste cell signal transmission without affecting sweet, bitter, and umami (TRPM5-positive) taste cell function.

We identified seven genes predicted or known to encode multi-transmembrane domain proteins expressed in specific subsets of macaque taste bud cells and confirmed expression of these genes in human taste buds. Localization of transcripts to TRPM5, PKD1L3, and SHH taste cell populations suggests the possible functions of these gene products in taste. Identification of gene products with links to calcium signalling (including ANO7, CALHM1-3, and MCTP1) in taste receptor cells highlights the central role of calcium in gustation. Future genetic and functional studies will illuminate the specific roles of these proteins in various taste processes including tastant recognition, signal transduction, and information coding to gustatory nerve fibers.

## Materials and Methods

### Taste Tissue

All primate samples (*Macaca fascicularis*) were collected in compliance with applicable federal, state, and local laws and regulations (CFR 1985 and PHS 1996) according to IACUC recommendations and oversight at both Charles River and Covance as previously described [7]. All human samples were collected with full written consent and with the approval of the Zoion Diagnostics institutional review board (IRB), an external IRB, and the IRB or Human Studies Committee at the organizations involved with the collection as previously described [7].

### Microarrays, Selection of Genes Encoding Multi-Transmembrane Proteins, and qPCR

Microarray-based gene expression data from lingual epithelium (LE), FG taste buds, CV taste buds, and the top and bottom fractions of CV taste buds was obtained from our primate taste bud gene expression database [7]. Statistical comparisons were made using unpaired, two-tailed Student's *t*-tests. Starting from the taste bud gene expression database available on-line (Supplementary Table 2 in [7]), we used Microsoft Excel to filter on the following functional descriptors in Column M: ‘multi-TM’ (80 genes), ‘channel’ (48 genes), ‘receptor’ (43 genes), ‘vesicular’ (12 genes), and ‘transport/transporter’ (87 genes). This resulted in a list of 270 genes, the majority of which are predicted to encode for multi-transmembrane proteins. ISH was performed for all genes as described below to identify genes expressed in a subset of taste cells, indicative of function in a particular taste cell type(s). From these analyses, we identified TMEM44, CALHM1, CALHM2, CALHM3, MCTP1, ANO7 (‘multi-TM’ descriptor), and SV2B (‘vesicular’ descriptor) as genes of interest.

Human RNA was isolated and cDNA was amplified from CV taste buds and lingual epithelium collected by laser capture microdissection from three donors as previously described [7]. Semi-quantitative PCR was performed using the Mx3000P® QPCR System (Stratagene, La Jolla CA) using TaqMan Universal PCR Master Mix (Applied Biosystems, Foster City, CA), 10 ng of cDNA, and the following inventoried TaqMan assays: TRPM5 (Hs00175822_m1), PKD2L1 (Hs00175850_m1), ANO7 (Hs00417639_m1), CALHM1 (Hs00736332_m1), CALHM3 (Hs00699323_m1), MCTP1 (Hs00226801_m1), SV2B (Hs00208178_m1), TMEM44 (Hs00961693_m1), and GAPDH (Hs99999905_m1). Relative expression (2^(40-Ct)^) was determined from analysis of duplicate samples [57]. A C_t_ value of 40 or more indicates no detectable transcript expression.

### Histology

*in situ* hybridization was performed as previously described [7], [33], [58]. Because the human and macaque genomes are ∼95% homologous [59], we hybridized human riboprobes to macaque tissue. Riboprobes were generated for TRPM5 (human: NM_014555; nt 396–2,006 and mouse: NM_ 020277 nt 2,293–3,447), PKD2L1 (human: NM_016112; nt 384–1,214 and mouse: NM_181422 nt 838–2,417), PKD1L3 (NM_181536; nt 1–1,079), TMEM44 (NM_138399; nt 170–1,489), SHH (NM_000193; nt 147–1,238), MCTP1 (human: NM_024717, nt 1,356–2,215 and mouse: NM_030174; nt 1,092–2,291), CALHM1 (NM_001001412; nt 755–2230), CALHM2 (NM_015916; nt 819–1,762), CALHM3 (NM_001129742; nt 939–1610), ANO7 (human: NM_001001891; nt 431–2195 and mouse: NM_207031; nt 179–1761), and SV2B (NM_014848; 1,076–2,635 nt). CALHM1, 2, and 3 probes were approximately 40% homologous and do not cross-hybridize under these ISH conditions. ISH studies were performed with two to three taste tissue samples from separate animals. Keratin-19 was stained using immunohistochemistry as previously described [33] following ISH. Keratin-19 monoclonal antibody (anti-cytokeratin 4.62; Sigma) is immunospecific for keratin-19 and has been previously used to label taste cells [11].

Digoxigenin and fluorescein labeled riboprobes were used to detect expression of two different genes in taste bud cells. Signals were developed using either colorimetric-fluorescent or fluorescent-fluorescent detection methods. For colorimetric-fluorescent detection, fluorescein-labeled riboprobes were first developed with peroxidase-conjugated anti-fluorescein antibody with tyramide signal amplification (TSA)-Cy3 (Perkin Elmer, Waltham, MA) and digoxigenin-labeled riboprobes were subsequently developed with alkaline phosphataste-conjugated anti-digoxigenin antibody (Roche, Indianapolis, IN) with NBT/BCIP substrate. For fluorescent-fluorescent detection, fluorescein-labeled riboprobes were first developed with peroxidase-conjugated anti-fluorescein antibody with TSA-Cy3 and digoxigenin-labeled riboprobes were subsequently developed with peroxidase-conjugated anti-digoxigenin antibody with TSA-FITC (Perkin Elmer). After the first TSA reaction, peroxidase activity was quenched with 3% hydrogen peroxide for 1 hr [60]. As a consequence of the staining process, colorimetric ISH signals (alkaline phosphatase-based chemistry) highlight the nuclear envelope and cytoplasm whereas fluorescent ISH signals (peroxidase-based chemistry with TSA amplification) highlight intranuclear regions [7], [60]; therefore, colocalization of colorimetric and fluorescent signals in the same cell results in the colorimetric signal encircling the fluorescent signal, whereas colocalization of two fluorescent signals in the same cell results in the signals overlapping (yellow pixels) in merged images. Double colorimetric-fluorescent ISH data were used to quantitate expression of transcripts in specific taste cell types since fluorescent signals could be more readily assigned to individual nuclei. Control hybridizations with sense riboprobes demonstrated signal specificity, and fluorescent-fluorescent detection with only a fluorescein-labeled riboprobe demonstrated complete peroxidase quenching.
